# Capsaicin Induces Apoptosis in KSHV-Positive Primary Effusion Lymphoma by Suppressing ERK and p38 MAPK Signaling and IL-6 Expression

**DOI:** 10.3389/fonc.2019.00083

**Published:** 2019-02-19

**Authors:** Misato Moriguchi, Tadashi Watanabe, Ayano Kadota, Masahiro Fujimuro

**Affiliations:** Department of Cell Biology, Kyoto Pharmaceutical University, Kyoto, Japan

**Keywords:** capsaicin, ERK, KSHV, hIL-6, p38 MAPK, PEL

## Abstract

Primary effusion lymphoma (PEL) is defined as a rare subtype of non-Hodgkin's B-cell lymphoma which is caused by Kaposi's sarcoma-associated herpesvirus (KSHV) in immunosuppressed patients. PEL is an aggressive lymphoma and is frequently resistant to conventional chemotherapies. Therefore, it is critical to investigate novel therapeutic options for PEL. Capsaicin is a pungent component of chili pepper and possesses unique pharmacological effects, such as pain relief, anti-microbial and anti-cancer properties. Here, we demonstrate that capsaicin markedly inhibited the growth of KSHV latently infected PEL cells by inhibiting ERK, p38 MAPK and expression hIL-6, which are known to contribute to PEL growth and survival. The underlying mechanism of action by capsaicin was through the inhibition of ERK and p38 MAPK phosphorylation and signaling that affected hIL-6 expression. As a result, capsaicin induced apoptosis in PEL cells in a caspase-9 dependent manner. In line with these results, ERK (U0126) and p38 MAPK (SB203580) specific signaling inhibitors suppressed hIL-6 expression and attenuated cell growth in PEL cells. Furthermore, the addition of hIL-6 neutralizing antibody to culture medium suppressed the growth of PEL cells. We also demonstrate that capsaicin suppressed PEL cell growth in the absence of nascent viral replication. Finally, we confirmed *ex vivo* treatment of capsaicin attenuated PEL development in SCID mice. Taken together, capsaicin could represent a lead compound for PEL therapy without the risk of *de novo* KSHV infection.

## Introduction

Primary effusion lymphoma (PEL) is classified as non-Hodgkin's B-cell lymphoma when it develops in immunocompromised patients, such as those with acquired immune deficiency syndrome (AIDS) patients, and those taking immunosuppressant drugs after having undergone organ transplantation (1, 2). PEL is usually treated with CHOP (cyclophosphamide, doxorubicin, vincristine, and prednisolone) therapy, which is frequently combined with rituximab; however, its prognosis is very poor (3). Therefore, it is an important issue in PEL treatment to develop more effective remedies. PEL cells are latently infected with Kaposi's sarcoma-associated herpesvirus (KSHV), which is also known as human herpesvirus-8 (HHV8). KSHV is a member of the γ-herpesvirus subfamily and is an oncogenic DNA virus that causes Kaposi's sarcoma, PEL, and multicentric Castleman's disease. Similar to other human herpesviruses, KSHV exists in either a latent or a lytic infection state, which can sustain the KSHV genome in the host for a prolonged period of time after the primary infection. During latency, the KSHV genome circularizes into a double-stranded episome and persists in the nucleus. In KSHV-infected cells including PEL cells, KSHV expresses LANA, viral FLIP, microRNAs, and small amounts of viral interleukin 6 (vIL-6) and viral IRFs (vIRFs), all of which strongly contribute to persistent infection or exert a malignant phenotype (4–6). These viral transcripts utilize and mimic cellular signaling, including NFκB, Wnt, p53, mitogen-activated protein kinases (MAPKs) and cytokine signaling. These KSHV-mediated disruptions dysregulate proliferation, apoptosis, immune escape and production of cytokines such as human interleukin 6 (hIL-6), IL-10 and vascular endothelial growth factor (VEGF) (4, 7–10). In particular, ERK and p38 MAPK signaling pathways are constitutively and/or transiently activated in PEL cells, which is necessary to establish KSHV primary infection (9, 10), transition from latent to lytic infection (4, 9, 10), and enhance the proliferation of PEL cells (4, 11–13). ERK1/2 is often phosphorylated by MEK1/2. The phosphorylated (i.e., activated) ERK1/2 can phosphorylate p90RSK and promote cell proliferation, survival, and metastasis through targeted gene expression (14). p38 MAPK is activated by cellular stress stimuli or cytokines and is associated with stress response, cytokine production, cell growth and apoptosis (15). p38 MAPK and ERK signaling further correlate with the production of inflammatory cytokine hIL-6 (16–21), which enhances cell proliferation of B-cell lymphoma including PEL (4, 22–24).

Capsaicin is a pungent component of chili pepper and exhibits several biological functions, such as pain relief and anti-bacterial effects (25–27). Furthermore, capsaicin exerts anti-cancer activity against several cancers, including breast, pancreatic, colon, epithelial and gastric cancers. These activities are believed to be due to the capsaicin-induced activation of p53 and ASK-1 or the capsaicin-induced inhibition of NADH oxidase, Wnt signaling, EGFR-HER2 signaling and MAPKs signaling (28–33). However, the effects of capsaicin on PEL remain largely unknown. Therefore, we investigated whether capsaicin induces growth inhibition of PEL cells and examined the underlying molecular mechanism involved.

## Materials and Methods

### Agents, Cell Lines, and Cell Culture

Capsaicin (*N*-[(4-hydroxy-3-methoxyphenyl) methyl]-8-methyl-6-nonenamide) (Wako, Osaka, Japan), U0126 (MEK inhibitor; LC Laboratories, Woburn, MA, USA), and SB203580 (p38 MAPK inhibitor; Nacalai Tesque Inc., Kyoto, Japan) were dissolved in dimethyl sulfoxide (DMSO). KSHV-positive PEL cell lines (BCBL1, BC2, BC3, HBL6, and JSC1) were kindly provided by Dr. S. D. Hayward (Johns Hopkins University School of Medicine, Baltimore, Maryland, USA). PEL and KSHV-negative lymphoma cell lines (Ramos, Raji, and DG75) were cultured in RPMI-1640 medium supplemented with 10% fetal bovine serum (FBS) (11). Ramos, Raji, and DG75 are classified into Burkitt's lymphoma, and Raji is EBV positive. Peripheral blood mononuclear cells (PBMCs) were separated from whole blood of ddY mice (Japan SLC, Inc., Shizuoka, Japan) by centrifugation through Histopaque-1083 (Sigma-Aldrich, Missouri, USA) and the cells from white buffy coat were washed two times with PBS and pellets were suspended in RPMI 1640 medium supplemented 30% inactivated FBS, 100 U/mL penicillin and 100 μg/mL streptomycin (Nacalai).

### Cell Viability Assay

Cells (1–4 × 10^4^ cells/well) were seeded onto 96-well plates. After incubation for 2 h, cells were treated with or without each compound at the indicated concentration for 24 h. The viable cell number was determined using Cell Count Reagent SF (Nacalai Tesque Inc.) as previously described (11). The optical density of each sample was measured at 450 nm on a microplate spectrophotometer (Tecan M200; Tecan, Kanagawa, Japan) and is expressed as a percentage (absorbance of untreated cells was defined as 100%).

### Two-Layered Soft Agar Colony Formation Assay

For soft agar assay, experiments were carried out in 12 well plates coated with a base layer of RPMI1640 containing 0.5% agar, 20% fetal bovine serum and capsaicin or vehicle. 5 × 10^3^ BCBL1 cells were seeded per well in RPMI1640 containing 0.35% agar, 20% fetal bovine serum and capsaicin (or vehicle) for 7 days. Colonies were visualized using a stereomicroscope (Olympus SZ61), and the number of colonies in a visual field was counted.

### Sodium Dodecyl Sulfate Polyacrylamide Gel Electrophoresis (SDS-PAGE) and Western Blot Analysis

SDS-PAGE and Western blot analysis were performed as previously described (11). For sample preparation, cells were solubilized in SDS sample buffer containing 5% 2-mercaptoethanol, 0.5 mM NaF, 0.5 mM β-glycerophosphate, and 0.1 mM PMSF. The primary antibodies used in this study were as follows: anti-Thr202/Tyr204-phospho-ERK1/2 (4370), Tyr705-phospho-STAT3 (9145), Ser217/221-phospho-MEK1/2 (9154), Ser380-phospho-p90RSK (11989), cleaved poly (ADP-ribose) polymerase (PARP) (9541), caspase-7 (9492), phospho-IKK-α/β (2697) (Cell Signaling Technology, Beverly, MA, USA); anti-Thr180/Tyr182-phospho-p38 MAPK (612288), p38 MAPK (612168), panERK (610123), STAT3 (610189), p90RSK (610225), GRP78 (610979), IκBα (610691), p65NFκB (610869), β-catenin (610154), MEK1 (610121), MEK2 (610235), BAD (610391), HSP90 (610419) (BD Biosciences, Franklin Lakes, NJ, USA); anti-β-actin (sc-69879) (Santa Cruz Biotechnology, Dallas, TX, USA).

### Caspase Assay

Cells (4 × 10^5^/mL) were incubated with 150 μM capsaicin or vehicle for 5 h, and the activities of caspase-3/7, -8, and -9 were measured using the Caspase-Glo Assay with a luciferin-conjugated polypeptide substrate (Promega, Madison, WI, USA) (13). Luminescence was measured on a luminescence microplate reader (Tecan M200). The caspase activities of untreated cells were defined as 1.0 relative light unit.

### Real-Time Reverse Transcription (RT) Polymerase Chain Reaction (PCR)

Real-time RT PCR was performed as previously described (11). Total RNA was purified and extracted from 1 × 10^6^ cells using RNAiso Plus (Takara Bio Inc., Shiga, Japan). First-strand cDNA was synthesized from 80 ng of total RNA using the ReverTra Ace qPCR RT Kit (Toyobo, Osaka, Japan), and real-time PCR was performed with THUNDERBIRD SYBR qPCR Mix (Toyobo) using the primer sets shown in Table 1. GAPDH was used as an internal control. The expression level of each gene was normalized to that of GAPDH.

**Table 1:** Primers for real-time PCR.

**Gene**	**Forward**	**Reverse**
hIL-6	5′-GGCTGAAAAAGATGGATGCTTC-3′	5′-TTTCTGCAGGAACTGGATCAG-3′
IL-10	5′-TCCCTGTGAAAACAAGAGCAAG-3′	5′-ATAGAGTCGCCACCCTGATG-3′
VEGF	5′-CACTGAGGAGTCCAACATCAC-3′	5′-GGTCTGCATTCACATTTGTTGTG-3′
vIL-6	5′-GGTCGGTTCACTGCTGGTATC-3′	5′-ATGCCGGTACGGTAACAGAG-3′
GAPDH	5′-TGACCACAGTCCATGCCATC-3′	5′-GGGGAGATTCAGTGTGGTGG-3′
ORF50	5′-ATAATCCGAATGCACACATCTTCCACCAC-3′	5′-TTCGTCGGCCTCTCGGACGAAACTGA-3′

### Cell Viability Assay in the Presence of hIL-6 Neutralizing Antibody

5 × 10^3^ cells were seeded onto 96-well plates and cultured in fresh RPMI medium supplemented with 10% FBS for 2 h. Anti-hIL-6 (554543, BD Biosciences) rat monoclonal antibody (mAb) or control rat IgG (Santa Cruz Biotechnology) was added into media (final concentration: 0.5 μg/mL), and cells were cultured for 24 h. The viable cell number was measured using Cell Count Reagent SF as described previously.

### Cell Viability Assay in the Presence of Recombinant hIL-6

1 × 10^4^ cells were washed twice by serum-free RPMI medium. Washed cells were re-suspended with medium containing 10% FBS and seeded onto 96-well plate. Recombinant hIL-6 (094-06043, Wako) was added into media (final concentration: 10 ng/mL), and cells were cultured for 0–3 days. The viable cell number was measured using Cell Count Reagent SF.

### Measurement of Virus Production

BCBL1 cells were cultured in media containing sodium butyrate (NaB; Tokyo Chemical Industry, Tokyo, Japan) with or without the test compound for 48 h to induce the lytic cycle and viral production (34). Culture supernatants were harvested and treated with DNase I (NEB, MA, USA) to obtain only enveloped and encapsidated viral genomes. Viral DNA was purified and extracted from culture supernatants using the QIAamp DNA Blood Mini Kit (QIAGEN, Valencia, CA, USA). To quantify viral DNA copies, SYBR Green real-time PCR was performed using KSHV-encoded ORF50 primers (Table 1).

### Animal Experiments

C.B-17 IcrHsd-Prkcd SCID male mice aged 5 weeks were purchased from Japan SLC, Incorporated. Mice were maintained at a 12 h light/dark cycle, and allowed to feed *ad libitum* on laboratory chow and water. Then mice were randomly divided into two groups (*n* = 4), and injected intraperitoneally with 250 μM capsaicin or vehicle treated-3.5 × 10^6^ BCBL1 cells in 200 μL PBS on day 0 (average body weight for each group was 20.48 g ± 0.64 and 20.67 g ± 0.57, respectively on day 0). Mice were observed and body weight was measured each day for 3 weeks. All mice were sacrificed on day 21, and the ascites were collected. The ascites collected from each mouse was centrifuged to determine the tumor volume.

All animal experiments were carried out in accordance with the Code of Ethics of the World Medical Association (Declaration of Helsinki) and the guiding principles for the care and use of laboratory animals in Kyoto Pharmaceutical University (KPU). Animal studies were approved by the Institutional Animal Use and Care Committee at KPU.

### Indirect Immunofluorescence Assay (IFA)

Ascites cells or BCBL1 cells treated with capsaicin or vehicle for 6 h were fixed on glass slides in 4% paraformaldehyde and permeabilized by 0.25% Triton X-100/PBS. Then it was blocked by 1% BSA/PBST and treated with each primary antibody and secondary antibody. DAPI was stained using Fluoro-KEEPER Antifade Reagent, Non-Hardening Type with DAPI (Nacalai). Anti-LANA antibody was established in our laboratory.

### Densitometry and Statistical Analyses

**Figure 1:**
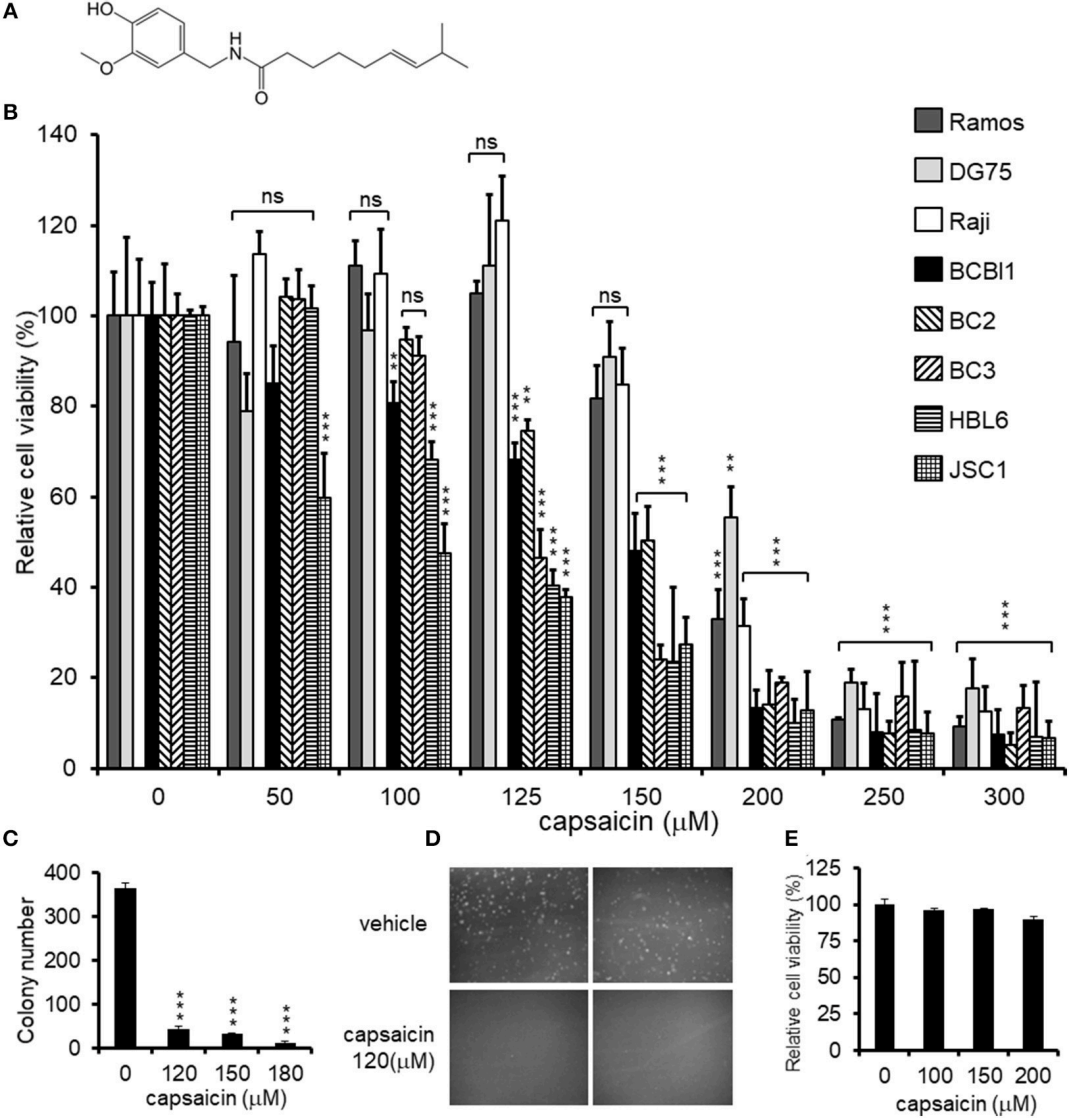
Cytotoxic effects of capsaicin on PEL and KSHV-uninfected B-lymphoma cells. **(A)** Chemical structure of capsaicin. **(B)** Cytotoxic effects of capsaicin on KSHV-positive PEL and KSHV-negative B-cell lines. PEL cells (BCBL1, BC2, BC3, HBL6, and JSC1) and KSHV-negative cells (Ramos, DG75, and Raji) were incubated with the indicated concentration of capsaicin for 24 h and the cell viability was assessed. The cell viability of the respective untreated cells was defined as 100%. ^**^P < 0.001 and ^***^P < 0.0001 indicate a statistically significantly difference compared with untreated cells. ns, not significant. **(C,D)** Soft agar colony formation assay. BCBL1 cells were cultured in soft agar containing 20% FBS and RPMI1640 with varying concentrations of capsaicin for 7 days. Colony formation was visualized using a stereomicroscope **(D)**, and the number of colonies per visual field was counted **(C)**. ^***^P < 0.0001 indicates a statistically significantly difference compared with untreated cells. **(E)** Cytotoxic effects of capsaicin on PBMCs. PBMCs from ddY mice were treated with varying doses of capsaicin for 24 h and cell viability was measured. The cell viability of the untreated cells was defined as 100%.

**Figure 2:**
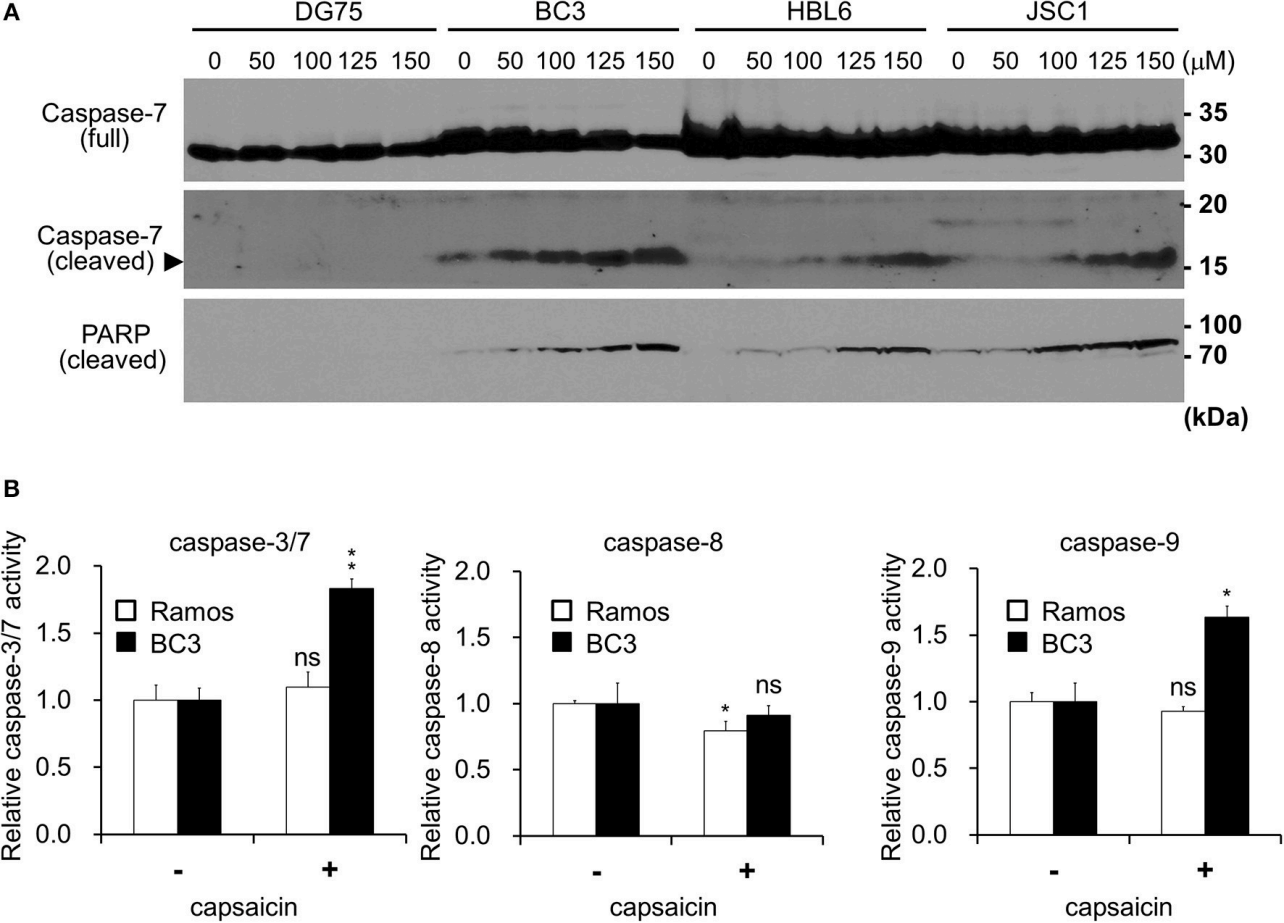
Capsaicin induces PEL apoptosis via caspase-9 activation. **(A)** Immunoblotting using antibodies against caspase-7 and cleaved-PARP. KSHV-infected PEL cells (BC3, HBL6, and JSC1) and KSHV-uninfected DG75 cells were incubated with the indicated concentration of capsaicin for 12 h and subjected to Western blot analysis. Arrowhead indicates cleaved caspase-7. **(B)** Changes in the proteolytic activities of caspase-3/7, -8, and -9 in PEL cells treated with capsaicin. BC3 and Ramos cells were incubated with 150 μM capsaicin for 5 h, and cell lysates were subjected to the caspase assay using a luciferin-conjugated polypeptide substrate. Caspase activity in untreated cells is represented as 1.0 relative light unit (RLU). ^*^P < 0.01 and ^**^P < 0.001 indicate a statistically significantly difference compared with untreated cells. ns, not significant.

**Figure 3:**
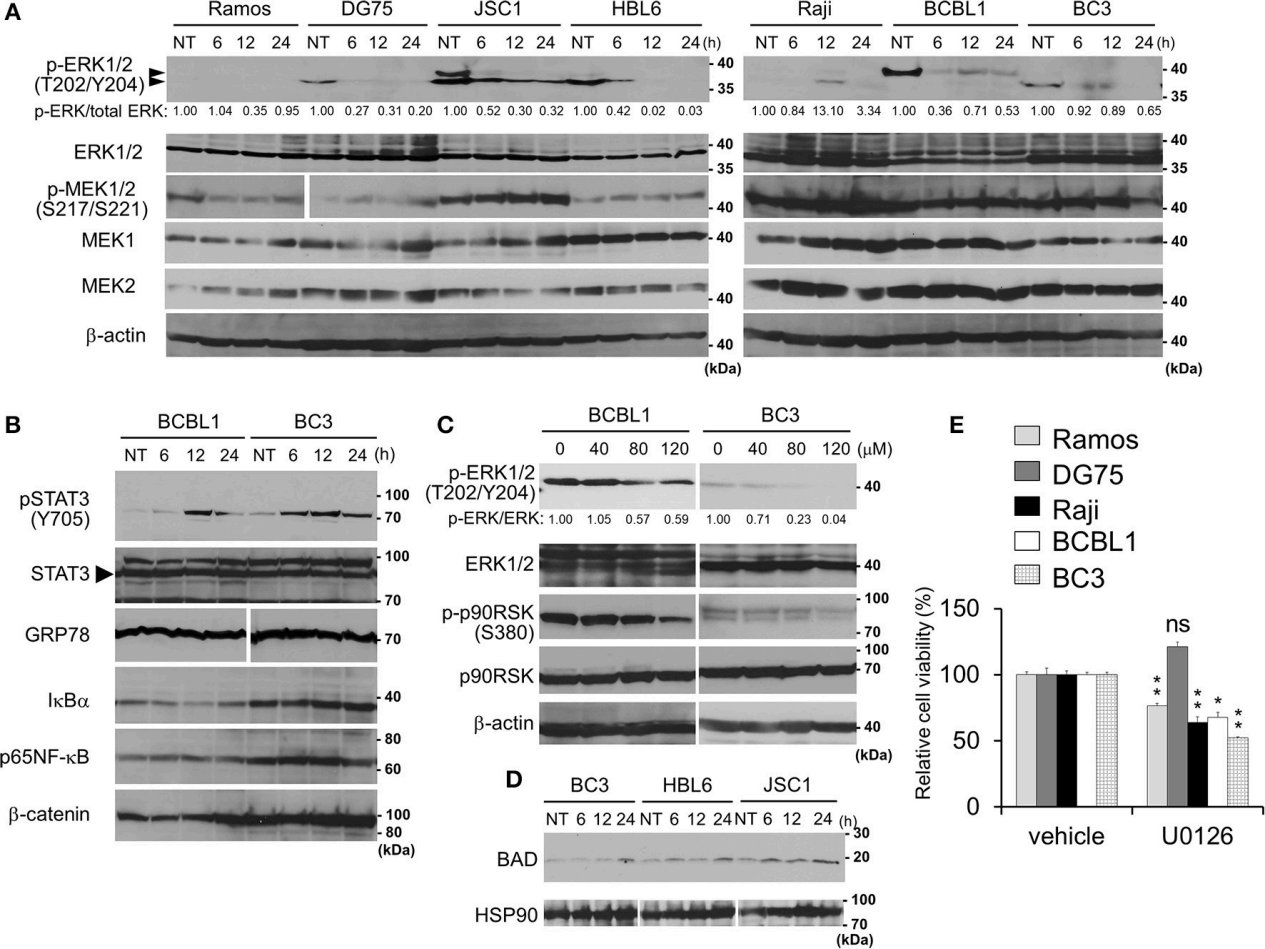
Capsaicin suppresses ERK signaling, which contributes to PEL growth. **(A)** Suppression of Thr202/Tyr204 phosphorylation of ERK1/2 by capsaicin in PEL cells. PEL (JSC1, HBL6, BCBL1, and BC3) and KSHV-negative (Ramos and DG75) cells were treated with 150 μM capsaicin for 6–24 h. Nontreated cells are denoted as NT. To investigate changes in the phosphorylation of ERK1/2 and MEK1/2, cell lysates were analyzed by Western blotting with anti-Thr202/Tyr204-phospho-ERK1/2, Ser217/221-phospho-MEK1/2, and panERK antibodies. The anti-Thr202/Tyr204-phospho-ERK1/2 antibody recognizes Thr202 and/or Tyr204 phospho-ERK1 (p44) and Thr185 and/or Tyr187 phospho-ERK2 (p42). Upper and lower arrowheads indicate phospho-ERK1 and phospho-ERK2, respectively. Band intensities of phospho-ERK were calculated using ImageJ software and normalized to those of total ERK. The values of phospho-ERK/total ERK are presented at the bottom of the picture, and the value of untreated cells is presented as 1.0. **(B)** Effects of capsaicin on the expression levels of phospho-STAT3, STAT3, GRP78, IκBα, p65NFκB, and β-catenin in BC3 and BCBL1 PEL cells. Cells were incubated with 150 μM capsaicin for 6–24 h and analyzed by Western blotting. Nontreated cells are denoted as NT. **(C)** Capsaicin suppressed the phosphorylation of ERK1/2 and p90RSK in a dose-dependent manner. BC3 and BCBL1 cells were incubated with the indicated concentration of capsaicin or vehicle (DMSO) for 24 h and subjected to Western blotting with anti-Thr202/Tyr204-phospho-ERK1/2, panERK, Ser380-phospho-p90RSK, and p90RSK antibodies. The intensity values of phospho-ERK/total ERK are presented at the bottom of the image, and the value of untreated cells is presented as 1.0. **(D)** Capsaicin increased pro-apoptotic Bcl family BAD. PEL (BC3, JSC1, and HBL6) cells were treated with 150 μM capsaicin for 6–24 h. Nontreated cells are denoted as NT. **(E)** U0126 (MEK inhibitor) suppresses growth of B-lymphomas except for DG75 cells. PEL (BC3 and BCBL1) and KSHV-uninfected (Ramos, DG75 and Raji) cells were treated with 100 μM U0126 or vehicle (DMSO) for 24 h. The values of the respective untreated cells were defined as 100%. ^*^P < 0.01 and ^**^P < 0.001 indicate a statistically significantly difference compared with untreated cells. ns, not significant.

**Figure 4:**
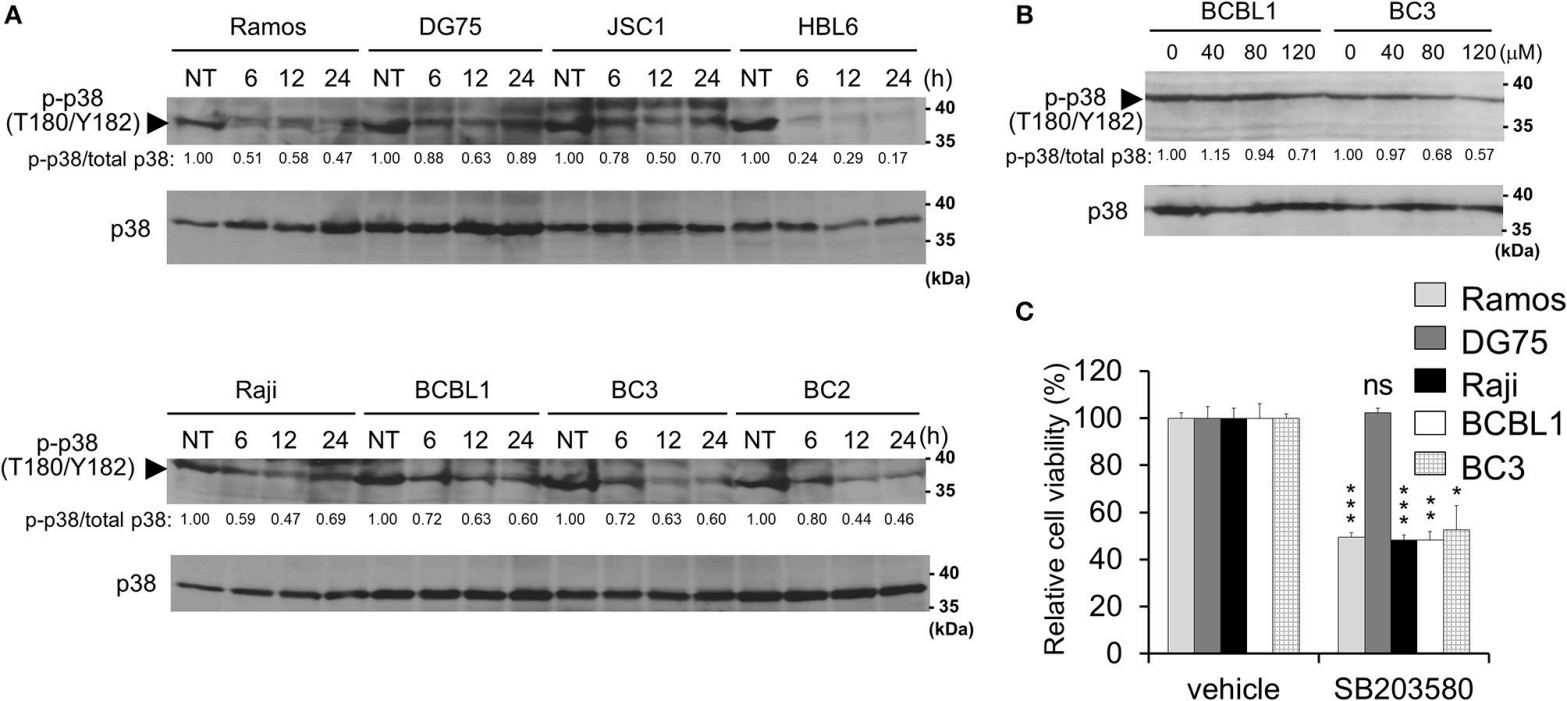
Capsaicin suppresses p38 MAPK signaling, which contributes to PEL growth. **(A)** Capsaicin decreases the Thr180/Tyr182 phosphorylation of p38 MAPK in PEL cells. PEL (JSC1, HBL6, BCBL1, BC3, and BC2) and KSHV-negative (Ramos, DG75, and Raji) cells were treated with 150 μM capsaicin and analyzed by Western blotting with anti-Thr180/Tyr182-phospho-p38 MAPK and p38 MAPK antibodies. Drug-untreated cells are denoted as NT. Arrowhead indicates phospho-p38 MAPK. The band intensities of phospho-p38 MAPK were normalized to those of total p38 MAPK. The values of phospho-p38 MAPK/total p38 MAPK are presented at the bottom of the image, and the value of untreated cells is presented as 1.0. **(B)** Capsaicin suppressed the p38 MAPK phosphorylation of PEL cells in a dose-dependent manner. Cells were incubated with the indicated concentration of capsaicin or vehicle (DMSO) for 24 h and analyzed by Western blotting with the anti-Thr180/Tyr182-phospho-p38 MAPK antibody. **(C)** SB203580 (p38 MAPK inhibitor) suppresses growth of B-lymphomas with the exception of DG75 cells. PEL (BC3 and BCBL1) and KSHV-negative (Ramos, DG75 and Raji) cells were treated with 100 μM SB203580 or vehicle (DMSO) for 24 h. The values of the respective untreated cells were defined as 100%. ^*^P < 0.01, ^**^P < 0.001, and ^***^P < 0.0001 indicate a statistically significant difference compared with untreated cells. ns, not significant.

**Figure 5:**
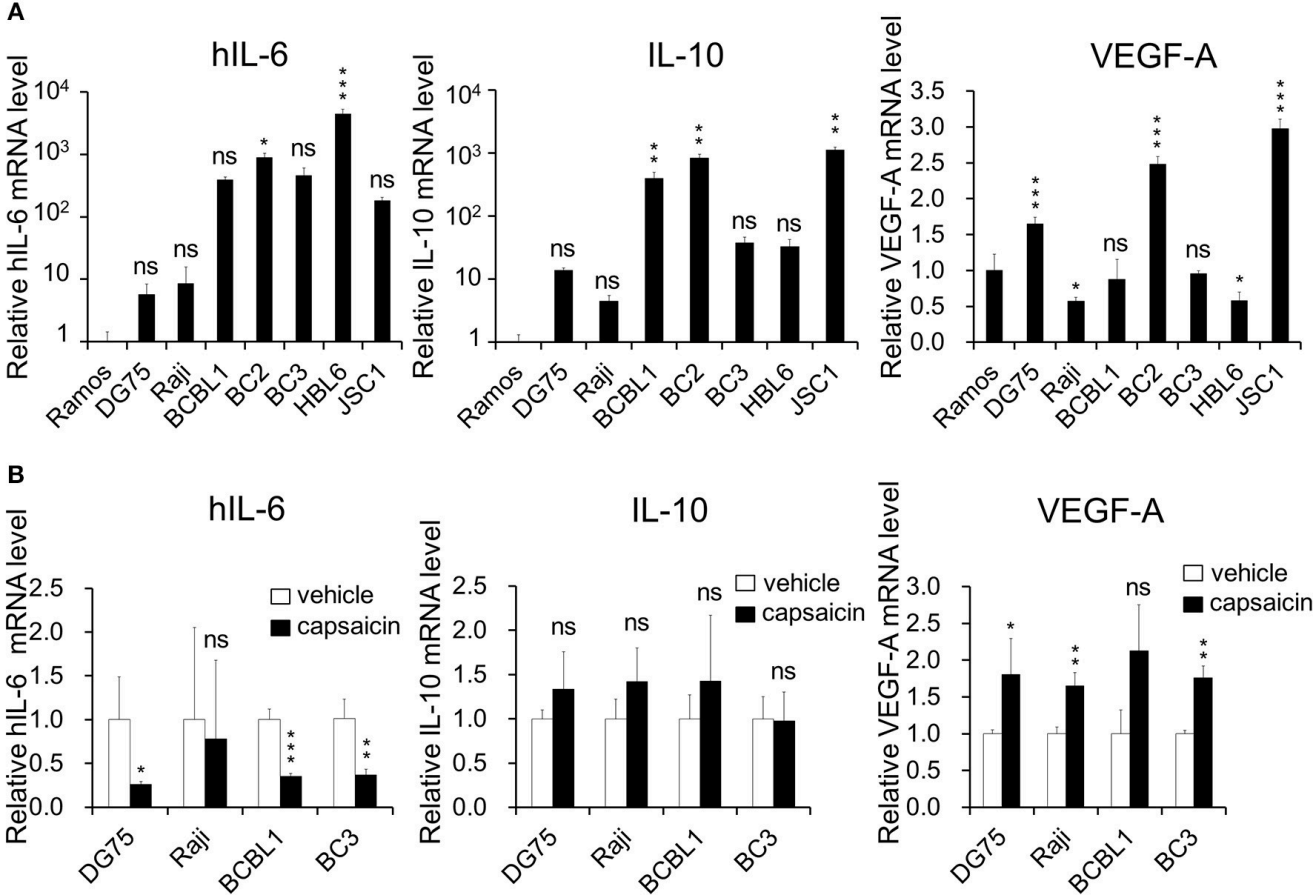
Expression of hIL-6 is upregulated in PEL cells and inhibited by capsaicin treatment. **(A)** The mRNA expression level of hIL-6 and IL-10 in B-lymphoma cells. Total RNA was extracted from PEL (BCBL1, BC2, BC3, HBL6, and JSC1) and KSHV-negative (Ramos, DG75, and Raji) cells, and purified RNA was subjected to RT-PCR to detect mRNA expression of hIL-6 and IL-10 and VEGF-A. The values obtained from Ramos cells were defined as 1.0. ^*^P < 0.1, ^**^P < 0.01, and ^***^P < 0.001 indicate a statistically significantly difference compared with untreated cells. ns, not significant. **(B)** The effects of capsaicin treatment on mRNA expression of hIL-6, IL-10 and VEGF-A. Cells were treated with or without 150 μM capsaicin for 3 h, and extracted total RNA was subjected to RT-PCR to quantitate the mRNA of hIL-6, IL-10, and VEGF-A. The values obtained from vehicle-treated cells were defined as 1.0. ^*^P < 0.1 and ^**^P < 0.01 indicate a statistically significantly difference compared with untreated cells. ns, not significant.

**Figure 6:**
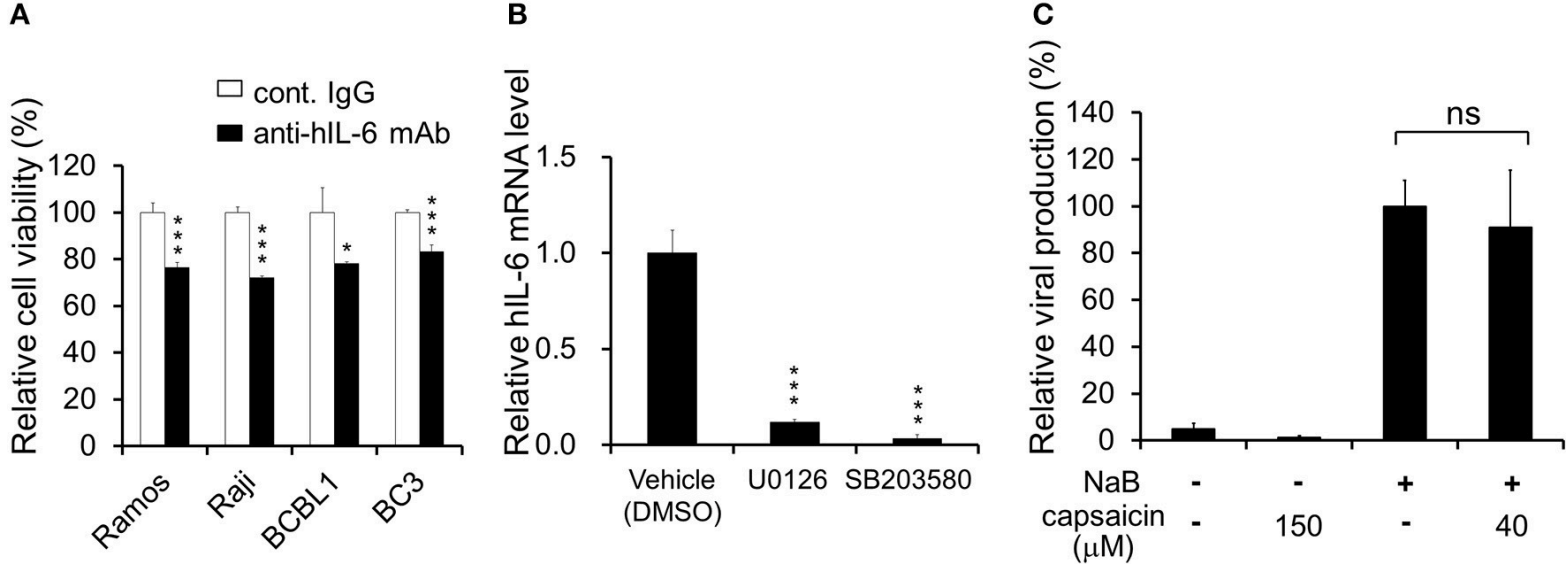
Neutralizing antibody against hIL-6 suppresses PEL proliferation, and inhibitors of ERK or p38 MAPK signaling downregulate hIL-6 expression. **(A)** Effect of hIL-6 neutralizing antibody on PEL growth. Cells were cultured in media containing anti-hIL-6 monoclonal antibody or control rat IgG and incubated for 24 h, and the cell viability was determined using Cell Count Reagent SF. The values obtained from control IgG-treated cells were defined as 100%. ^*^P < 0.1 and ^***^P < 0.001 indicate a statistically significantly difference compared with untreated cells. ns, not significant. **(B)** Effect of ERK1/2 or p38 MAPK signaling inhibitor on hIL-6 mRNA expression in BCBL1 cells. BCBL1 cells were treated with MEKL1/2 inhibitor (U0126) or p38 MAPK inhibitor (SB203580) for 3 h, and extracted total RNA was subjected to RT-PCR. The values obtained from vehicle-treated cells were defined as 1.0 ^***^P < 0.001 indicate a statistically significantly difference compared with untreated cells. ns, not significant. **(C)** Capsaicin does not influence viral production in PEL cells. Sodium butyrate (NaB) was used as an inducer of the lytic cycle. BCBL1 cells were cultured in media containing 150 or 40 μM capsaicin for 48 h. Culture media containing virus particles were harvested, and KSHV genomes were quantified by real-time PCR. The value of the viral genome in NaB-treated and capsaicin-untreated cells was defined as 100%. ns, not significant.

**Figure 7:**
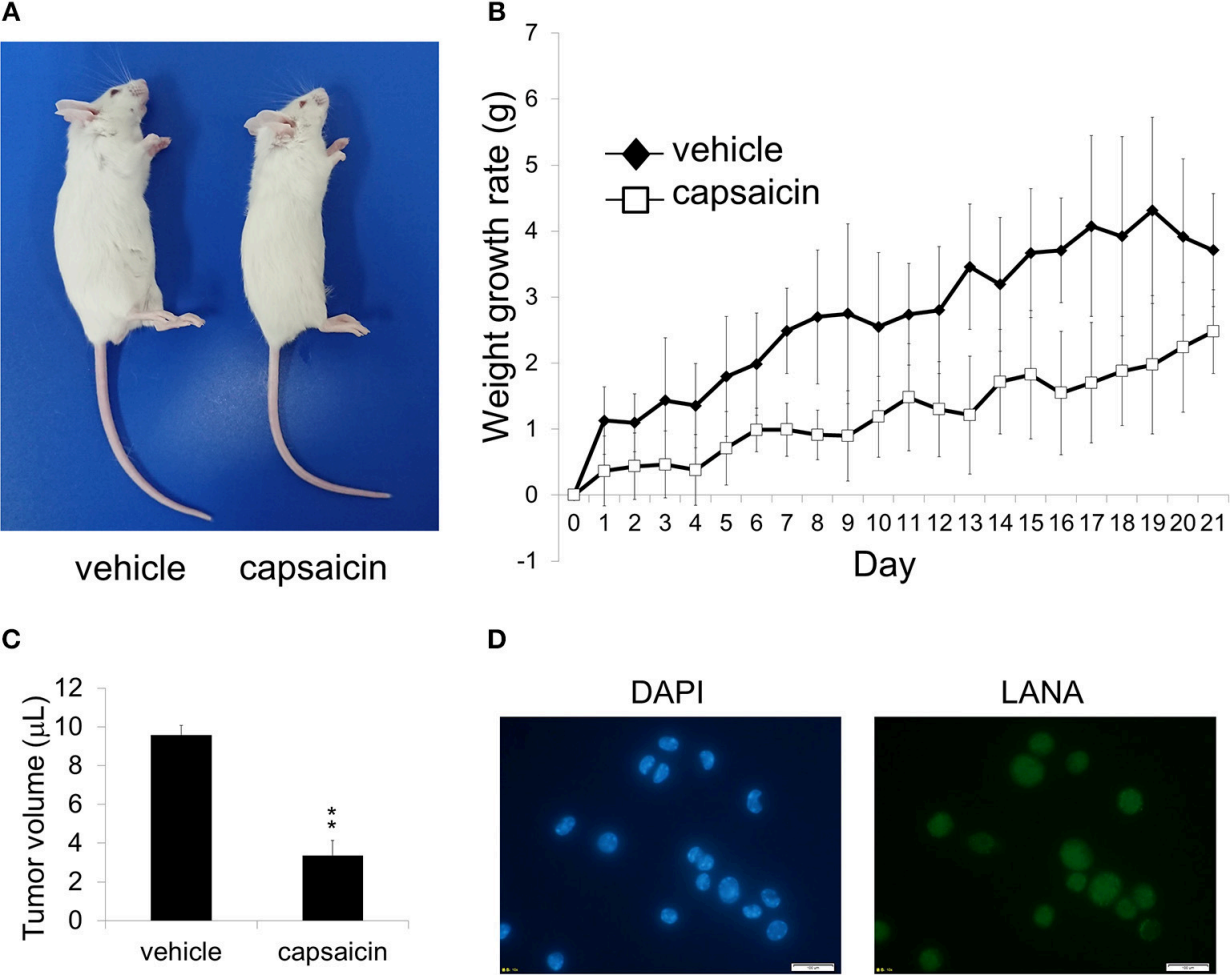
*Ex vivo* treatment of PEL cells with capsaicin suppressed their *in vivo* in SCID mice. **(A)** An image showing vehicle (DMSO)-pretreated BCBL1 cells injected (left) and capsaicin-pretreated BCBL1 cells injected (right) in SCID mice on day 21 after transplantation. SCID mice were injected intraperitoneally with 3.5 × 10^6^ BCBL1 cells treated with 250 μM capsaicin or vehicle for 6 h *ex vivo* on day 0 **(B)** The body weight changes of BCBL1 engrafted SCID mice for 3 weeks each day after grafting. The body weight changes of vehicle-pretreated (*n* = 4) are indicated by black rhombus, and those of capsaicin-pretreated (*n* = 4) are indicated by white squares. **(C)** The volume of intraperitoneal tumor cells from vehicle-pretreated and capsaicin-pretreated mice. The average of tumor volume from each mice (*n* = 3) was shown. ^**^P < 0.001 indicates a statistically significant difference compared with control. **(D)** LANA expression of ascites cells from mice transplanted with vehicle-treated BCBL1 was confirmed by IFA using anti-LANA antibody. The white bar indicates 100 μm.

Densitometric analysis of Western blots was performed using ImageJ software (NIH, Bethesda, MD, USA). The results were quantified in arbitrary units, where 1 represents the level of the drug-untreated control. The standard deviation was determined by analyzing the data from at least three experiments and is indicated by error bars. The statistical significance between each group and the control was analyzed by one-way analysis of variance followed by Dunnett's test for multiple comparisons (Figures 1B,C, **5A**) or the two-tailed Student's *t*-test (Figures 2B, 3E, 4C, 5B, 6A–C, 7C; Supplemental Figures S2, S3).

## Results

### Capsaicin Exhibits Cytotoxicity Against PEL Cells

First, we investigated the cytotoxic effect of various concentrations of capsaicin (Figure 1A) on KSHV-infected and KSHV-uninfected B-lymphoma cells (Figure 1B). KSHV latently-infected PEL cell lines (BCBL1, BC2, BC3, HBL6, and JSC1) and uninfected B-cell lines (Ramos, DG75, and Raji) were treated with the indicated concentration of capsaicin or vehicle (DMSO) for 24 h. Capsaicin inhibited the proliferation of PEL cell lines compared with uninfected B-cell lines. The viability of PEL cells was preferentially decreased compared to that of uninfected cells when treated with between 125 and 150 μM of capsaicin. The 50% cytotoxic concentration (CC_50_) values of capsaicin on the tested B-cell lines are summarized in Table 2. In addition, inhibitory effect of capsaicin on colony formation of BCBL1 PEL was elucidated. Each dose of capsaicin significantly decreased colony formation of BCBL1 cells compared with DMSO-treated cells (Figures 1C,D). We also checked the cytotoxic effect of capsaicin on peripheral blood mononuclear cells (PBMCs) and found that capsaicin did not affect the survival of normal mouse PBMCs (Figure 1E).

**Table 2:** Cytotoxic effects of capsaicin on B-lymphoma cell lines.

	**Ramos**	**DG75**	**Raji**	**BCBL1**	**BC2**	**BC3**	**HBL6**	**JSC1**
CC_50_ (μM)	182.6	207.6	182.6	147.6	150.6	114.2	116.4	90.3

*CC_50_, cytotoxic concentration of capsaicin that reduces cell viability by 50%*.

### Capsaicin Induces Caspase-9-Mediated Apoptosis in PEL Cells

Next, we examined whether the anti-proliferative effects of capsaicin are due to apoptotic cell death. PEL cells were treated with capsaicin for 12 h, and caspase-7 and PARP cleavage were examined by Western blotting (Figure 2A). Capsaicin promoted the cleavage of caspase-7 and PARP in PEL cells in a dose-dependent manner. We also measured the peptidase activities of caspase-3/7, -8, and -9 in capsaicin-treated cells (Figure 2B). After incubation with 150 μM capsaicin for 5 h, the activation of caspase-3/7 and -9 was detected in BC3 cells but not in Ramos cells. In contrast, caspase -8 activity was not increased in either cell line. These results indicate that capsaicin suppresses the growth of PEL cells by triggering apoptosis, which is mediated by caspase-9 activation, resulting in activation of the executioner caspases, caspase-3 and -7.

### Capsaicin Suppresses ERK Signaling in PEL

ERK, NFκB, STAT3, and Wnt signaling are known to be activated and associated with cell growth and survival of PEL (4, 7, 8). Therefore, we analyzed changes in these pathways in the presence of capsaicin (Figures 3A,B). PEL cells (JSC1, HBL6, BCBL1, and BC3) were incubated with capsaicin for 6–24 h and subjected to Western blotting. The levels of IκBα, p65NFκB, β-catenin, and GRP78 did not change, whereas phosphorylated STAT3 were increased in PEL cells (Figure 3B). We also examined the expression levels of these molecules in other B-lymphoma cells, including PEL (Supplemental Figure S1); remarkable changes were not detected. Moreover, we assessed whether capsaicin reduced the nuclear translocation of p65NFκB and phosphorylation of IKKα/β, however, capsaicin treatment did not change them (Supplemental Figures S2, S3). On the other hand, the phosphorylation of Thr202/Tyr204 in ERK1/2 was decreased in PEL cells by capsaicin treatment (Figure 3A), suggesting that capsaicin suppressed ERK signaling in PEL cells. However, inhibition of MEK1/2 phosphorylation by capsaicin was not observed in PEL cells. We also confirmed that capsaicin suppressed the phosphorylation of ERK1/2 and p90RSK (a downstream target of ERK1/2) in a dose-dependent manner (Figure 3C). In addition to p90RSK, BAD (another target of ERK1/2) was increased by capsaicin treatment (Figure 3D). It is known that a pro-apoptotic Bcl family, BAD, is phosphorylated by phospho-ERK1/2 and phosphorylated BAD is degraded. Because ERK signaling activation is necessary for the survival and proliferation of PEL cells (4, 11–13), we speculated that capsaicin-mediated ERK suppression exerts cytotoxic effects against PEL cells. Therefore, we examined the effect of U0126, a MEK1/2 inhibitor, on PEL and KSHV-negative cell proliferation. As a result, U0126 suppressed cell growth of these cells except for DG75 cells (Figure 3E). These results suggest that capsaicin suppressed ERK signaling in PEL cells, and this signaling was important for survival of B-lymphomas with the exception of DG75 cells, which correlated with Figure 1B.

### Capsaicin Suppresses p38 MAPK Signaling and Expression of hIL-6 mRNA

In addition to ERK, p38 MAPK signaling is also activated in PEL cells and is associated with PEL cell proliferation (9–11). Therefore, we investigated p38 MAPK phosphorylation in capsaicin-treated PEL cells. Capsaicin suppressed the phosphorylation of p38 MAPK in a time- and dose-dependent manner (Figures 4A,B). However, unlike ERK1/2, the dephosphorylation of p38 MAPK was detected in both PEL cells and uninfected B-cells (Ramos, DG75, and Raji). Next, to evaluate whether the anti-proliferative effects of capsaicin are due to capsaicin-mediated p38 MAPK inhibition, we assessed the cytotoxic effect of the p38 MAPK inhibitor (SB203580) on PEL and KSHV-uninfected cells (Figure 4C). SB203580 suppressed the growth of B-cells with the exception of DG75 cell.

ERK and p38 MAPK are known to strongly correlate with hIL-6 production (16, 19–21). Furthermore, expression and secretion of hIL-6, VEGF-A and anti-inflammatory cytokine IL-10 is increased in PEL cells and are associated with PEL proliferation (4, 22, 35, 36). Therefore, we examined the effects of capsaicin on mRNA levels of hIL-6, IL-10 and VEGF-A in PEL cells. hIL-6 and IL-10 were highly expressed in all PEL cells compared with KSHV-negative cells (Figure 5A). On the other hand, VEGF was increased in some PEL cells such as BC2 and JSC1. Interestingly, capsaicin treatment significantly decreased expression of hIL-6 mRNA but not those of IL-10 and VEGF in all tested B cells (Figure 5B). On the contrary, IL-10 and VEGF were increased by capsaicin. Next, we investigated the effects of supplementing exogenous recombinant hIL-6 or neutralizing extracellular hIL-6 on PEL proliferation. When cells were cultured in 10% FBS-containing media with recombinant hIL-6, proliferation of PEL and uninfected cells were not changed (Supplemental Figure S2). In contrast, neutralization of hIL-6 in media by anti-hIL-6 antibody suppressed proliferation of PEL and uninfected cells (Figure 6A), suggesting that the presence of some amount of extracellular hIL-6 is necessary for PEL growth and/or survival. Hence, we further investigated whether inhibition of ERK and p38 MAPK signaling decreases hIL-6 production in PEL. Results indicate that both of MEK1/2 inhibitor (U0126) and p38 MAPK inhibitor (SB203580) downregulated hIL-6 mRNA level in BCBL1 (Figure 6B). Viral interleukin-6 (vIL-6) is a viral homolog of hIL-6 and encoded by KSHV and is expressed in KSHV-associated cancer cells and to a higher extent during viral replication. In addition to real-time RT PCR detection, we also attempted to measure hIL-6 protein in cultue media by ELISA. However, we failed to detect hIL-6 due to the limited sensitivity of the assay. We also examined the effect of capsaicin on the vIL-6 expression level in PEL cells. however vIL-6 mRNA in BCBL1 cells was unchanged (Supplemental Figure S3). Collectively, these results indicate that capsaicin suppresses not only p38 MAPK signaling but also hIL-6 expression, which results in the suppression of PEL growth.

### Effects of Capsaicin on KSHV Production in PEL

In the lytic replication, KSHV virions are produced in PEL cells and released, resulting in cell death. As treatment with over 125 μM capsaicin significantly decreased cell viability of PEL cells, we clarified whether capsaicin induces lytic replication in KSHV latently infected PEL (Figure 6C). In addition, we evaluated whether capsaicin suppresses the lytic replication. Sodium butyrate (NaB), an inducer of the lytic cycle, efficiently induced viral production, whereas 150 μM capsaicin which suppressed BCBL1 cell growth (Figure 1B) did not. Furthermore, capsaicin did not suppress NaB-induced viral production at the low concentration (40 μM), which did not affect BCBL1 cell growth (Figure 1B). These results indicate that capsaicin does not influence the life cycle of KSHV in PEL cells but, instead, triggers PEL cell death in the absence of nascent viral replication and production.

### *Ex vivo* Treatment of Capsaicin Suppresses the Growth of PEL in SCID Mice

As capsaicin exhibited cytotoxicity on PEL cell lines *in vitro* (Figures 1B–D), we next assessed whether capsaicin suppressed PEL development in a mouse xenograft model. Before transplantation, BCBL1 cells were treated with capsaicin or vehicle for 6 h. Then the cells were washed and suspended with PBS, and injected intraperitoneally into SCID mice on day 0. The physical condition and weight development of the treated mice were observed until day 21. As a result, the abdomen of mice (vehicle-pretreated BCBL1 injected) showed enlargement (Figure 7A), and the body weight was increased (Figure 7B) compared with capsaicin-pretreated BCBL1 injected mice. Moreover, capsaicin inhibited growth of tumor volume in ascites (Figure 7C). We also confirmed that tumor cells in ascites collected from vehicle-pretreated mice were indeed infected with KSHV using IFA against LANA (a marker of latent KSHV infection) (Figure 7D). These results suggested that capsaicin treatment prevented BCBL1 development in mouse peritoneal cavities.

## Discussion

**Figure 8:**
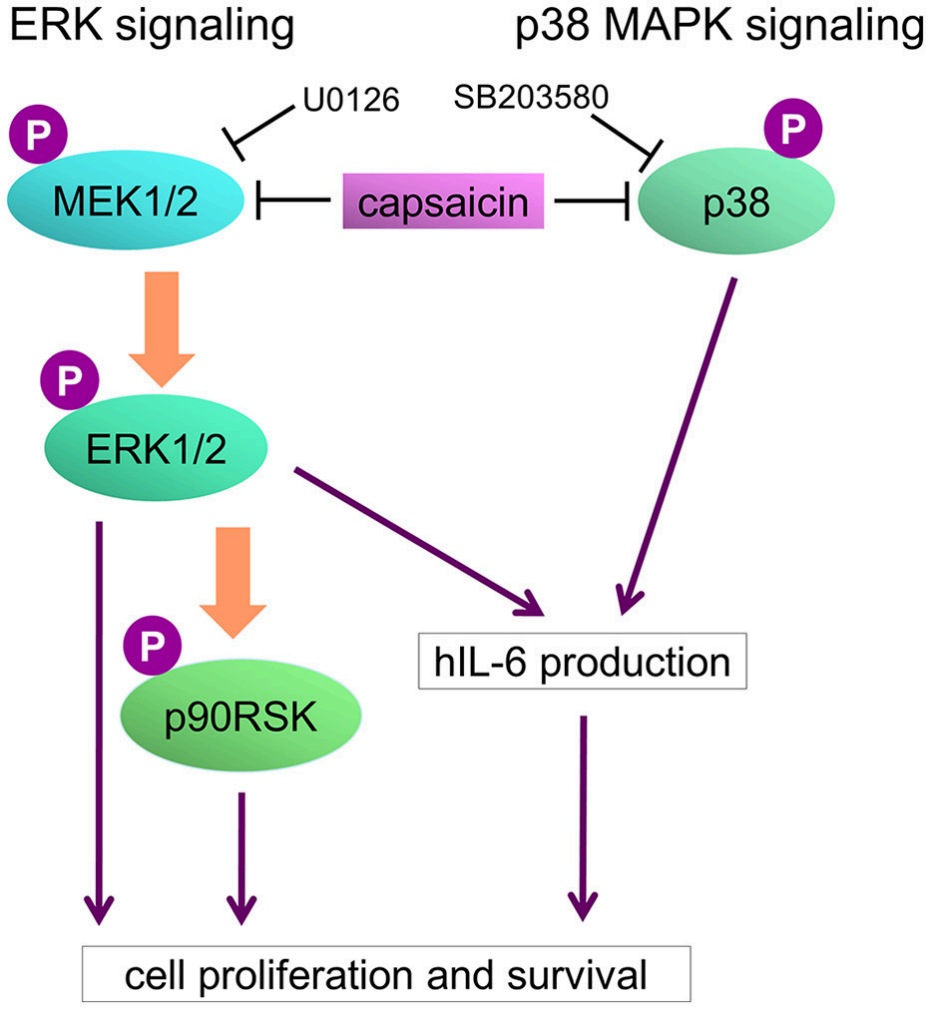
Model of capsaicin-mediated suppression of PEL growth. ERK and p38 MAPK signaling pathways are known to be necessary for PEL growth and hIL-6 expression. hIL-6 mRNA are highly expressed in PEL cells, and secreted extracellular hIL-6 also contributes to PEL growth. Capsaicin induces suppression of ERK and p38 MAPK signaling, resulting in hIL-6 downregulation and growth inhibition of PEL cells.

In this study, we discovered that capsaicin has preferential cytotoxic activity against PEL to induce apoptosis compared with KSHV-uninfected B-lymphoma cells. KSHV constitutively promotes ERK and p38 MAPK signaling and hIL-6 expression in PEL cells, which might be related with higher sensitivity of PEL cells to capsaicin. KSHV-negative cells were less sensitive to capsaicin likely owing to their independence from these signals for proliferation and survival. We propose a model in which capsaicin downregulates MAPKs and hIL-6 expression of PEL (Figure 8). KSHV is known to activate ERK and p38 MAPK signaling in the infected cells, allowing primary infection (9, 10), viral replication (4, 9, 10), proliferation of infected cells (4, 11–13), and hIL-6 expression (18, 19, 37). We found that capsaicin suppressed ERK1 and/or ERK2 phosphorylation in PEL cells, and ERK1/2 inhibition by U0126 suppressed PEL growth (Figure 3). However, capsaicin did not inhibit MEK1/2 phosphorylation in PEL cells, suggesting that capsaicin inhibits MEK1/2 or other unknown kinases that phosphorylate ERK1/2, resulting in the inhibition of ERK1/2 phosphorylation. While ERK1 and ERK2 have similar peptide sequences (84% identity), and their functions are substantially overlapping (38), it was reported that ERK2 affected some specific abilities such as learning and memory (39). In B cells, Expression of either ERK1 or ERK2 is required for proliferation (40). ERK signaling is well-known for its role in cell proliferation signaling, and is activated in many cancers including PEL (4, 9, 10). In fact, an ERK signaling inhibitor, trametinib, has been clinically and widely used to treat ERK-dependent cancers like malignant melanoma (41). We also previously reported that arctigenin, sangivamycin and C_60_ fullerene derivative induced PEL cell death by ERK inhibition (11–13). These lines of evidence imply that inhibition of ERK signaling causes suppression of PEL growth. In addition to ERK, we suppressed of p38 MAPK signaling and PEL cell growth by capsaicin (Figure 4). When p38 MAPK signaling is activated by cell stress and growth factors, this signaling induces the expression of transcriptional targets which are related to stress response, inflammation, proliferation and so on. p38 MAPK signaling is frequently activated in several cancers (42–44) including PEL (10, 11), and p38 MAPK inhibition induced cancer cell death (42, 43). We also reported arctigenin-mediated p38 MAPK inhibition and cytotoxic effect of arctigenin on PEL (11). These studies and our findings therefore support the idea that p38 MAPK as well as ERK, is a potential therapeutic target of PEL.

We have demonstrated that mRNA expression of hIL-6 and IL-10 was upregulated in PEL cells (Figure 5A), and hIL-6 expression is suppressed by capsaicin treatment (Figure 5B) and inhibition of ERK1/2 and p38 MAPK signaling (Figure 6B). Furthermore, extracellular hIL-6 neutralization by antibody treatment inhibited PEL proliferation (Figure 6A). It has been well known that hIL-6 was discovered as a B-cell proliferative factor (23, 24), and activation of ERK or p38 MAPK signaling enhances hIL-6 expression (16, 18–21, 37). Moreover, hIL-6 expression is reported to be upregulated in PEL cells (4, 22, 35). These reports are in agreement with our findings (Figures 5, 6). However, exogenously added hIL-6 in culture media did not further influence growth of PEL cells (Figure S2), suggesting that endogenously produced hIL-6 is sufficient for PEL proliferation. In support of this hypothesis, there are similar reports that exogenously added hIL-6 in culture media did not promote PEL proliferation (35, 45). On the other hand, hIL-6 knockdown or hIL-6 signaling inhibition experiments were reported to suppress cell growth of PEL. For example, antisense oligonucleotides to hIL-6 gene (22) or Tocilizumab (antibody against hIL-6 receptor alpha) (36) decreased PEL cell growth, which are in line with our result showing inhibition of PEL proliferation by hIL-6-neutralizing antibody (Figure 6A). Thus, because the presence of extracellular hIL-6 in culture medium strongly contributes to PEL growth or survival, we speculate that hIL-6 neutralizing antibody suppresses PEL growth.

Capsaicin has many pharmacological effects such as anti-cancer, pain relief, anti-microbial and anti-inflammation. Capsaicin was reported to induce apoptosis in various types of cancers by modulating signaling pathways such as p53 (28), Wnt (31), p38 MAPK (30, 46) and ERK (30, 33, 46). Regarding STAT3, there are reports, showing that capsaicin activates STAT3 signaling by STAT3 phosphorylation (47, 48), which are in agreement with our findings in capsaicin-treated PEL cells (Figure 3B and Supplemental Figure S1A). In addition, capsaicin induced mRNA expression of VEGF-A, which is a downstream target of STAT3 (Figure 5B). Granato et al. reported that capsaicin suppressed proliferation of BC3 and BCBL1 PEL by suppressing STAT3 activation (49). Thus, we consider that this difference may be due to different concentrations and/or durations of capsaicin treatment. In addition to signaling, Capsaicin is shown to stimulate transient receptor potential vanilloid-1 (TRPV1) and to cause pain (50), whereas high-dose capsaicin desensitizes sensory neurons and suppressed pain and inflammation (51). Furthermore, topical capsaicin patch reduced peripheral neuropathy in patients treated oxaliplatin-based chemotherapy (25). These indicate that capsaicin has other potential clinical benefits, besides anti-cancer activity.

In our first attempt to conduct *in vivo* animal studies, SCID mice xenografted with PEL cells were established, and capsaicin was administered into the intraperitoneal region. However, capsaicin administration immediately killed mice accompanied by convulsions. Therefore, we performed *ex-vivo* treatment of PEL cells with capsaicin before SCID mice were xenografted (Figure 7). We believe the death of the animals after direct capsaicin administration may be due to tumor lysis syndrome and excessive stimulation of the nervous system by the high dose of capsaicin administered. Further development of capsaicin derivatives, aimed at decreasing these side effects, need to be explored in future studies.

In conclusion, capsaicin suppresses ERK and p38 MAPK pathways in PEL cells, which results in downregulation of hIL-6 and subsequently apoptosis. These biological functions of capsaicin can serve as cytotoxic effectors in PEL cells. Therefore, PEL cells are more sensitive to the anti-proliferative effect of capsaicin than KSHV-uninfected cells, meaning that capsaicin may serve as an effective therapy for KSHV-associated cancers including PEL.

## Author Contributions

MM designed the experiments, performed most of the experiments and analyzed the results. TW established experiments using real-time PCR, performed the animal experiment, and analyzed the results. AK performed the animal experiment. MF designed the project, analyzed the results and prepared the manuscript.

### Conflict of Interest Statement

The authors declare that the research was conducted in the absence of any commercial or financial relationships that could be construed as a potential conflict of interest.

## Supplementary Material

Supplemental Figure S1 **(A)** Immunoblotting analysis of the phosphorylated STAT3, GRP78, IκBα, p65NFκB, and β-catenin in capsaicin-treated B-lymphoma cells. KSHV-negative cells (Ramos and DG75) and JSC1 PEL cells were treated with 150 μM capsaicin for 6–24 h, and whole-cell lysates were assayed by immunoblotting with anti-Tyr705-phospho-STAT3, STAT3, GRP78, IκBα, p65NFκB, or β-catenin antibodies. Nontreated cells are denoted as NT. Arrowhead indicates STAT3. **(B)** Subcellular localization of p65NFκB in capsaicin-treated (or untreated) BCBL1 cells. BCBL1 cells were treated with capsaicin or vehicle for 6 h, the p65NFκB (green) and DNA (blue) was detected by IFA. The white bar indicates 100 μm. **(C)** Capsaicin did not affect phospho-IKKα/β. PEL (JSC1, HBL6, BCBL1, and BC3) cells were treated with 150 μM capsaicin for 6–24 h, and whole-cell lysates were assayed by immunoblotting with anti-phospho-IKKα/β antibodies. Nontreated cells are denoted as NT.

Supplemental Figure S2 Effect of exogenously supplied hIL-6 on proliferation of B-lymphoma cells. Cells were treated with 10 ng/mL of recombinant hIL-6 and incubated for 0–3 days. The y-axis represents the hIL-6-dependent growth rate (the cell number of the hIL-6-treated cells vs. nontreated cells). Namely, the y-axis represents the percentage of hIL-6-treated cell number when the cell number of nontreated cells on each day is defined as 100%. ^^*^^*P* < 0.1 indicate a statistically significantly difference compared with untreated cells. ns, not significant.

Supplemental Figure S3 Capsaicin treatment does not affect mRNA expression of KSHV-encoded vIL-6. BCBL1 cells were treated with 150 μM capsaicin or vehicle for 3 h, and extracted total RNA was subjected to RT-PCR to quantitate mRNA of vIL-6. The values obtained from vehicle-treated cells were defined as 1.0. ns, not significant.

## Abbreviations

AIDS: acquired immunodeficiency syndrome
ERK: extracellular signal-regulated kinase
HHV8: human herpesvirus-8
KSHV: Kaposi's sarcoma-associated herpesvirus
hIL-6: human interleukine-6
vIL-6: viral interleukine-6
LANA: latency-associated nuclear antigen
p38 MAPK: p38 mitogen-activated protein kinase
MEK: mitogen-activated protein kinase kinase
PEL: primary effusion lymphoma
RSK: ribosomal protein S6 kinase
VEGF: vascular endothelial growth factor
v-IRFs: viral interferon regulatory factors.
