# Supplementary figures and images for: Capsaicin Induces Apoptosis in KSHV-Positive Primary Effusion Lymphoma by Suppressing ERK and p38 MAPK Signaling and IL-6 Expression
Source: Front Oncol. 2019 Feb 19;9:83. doi: 10.3389/fonc.2019.00083 (PMC6389641; doi:10.3389/fonc.2019.00083)

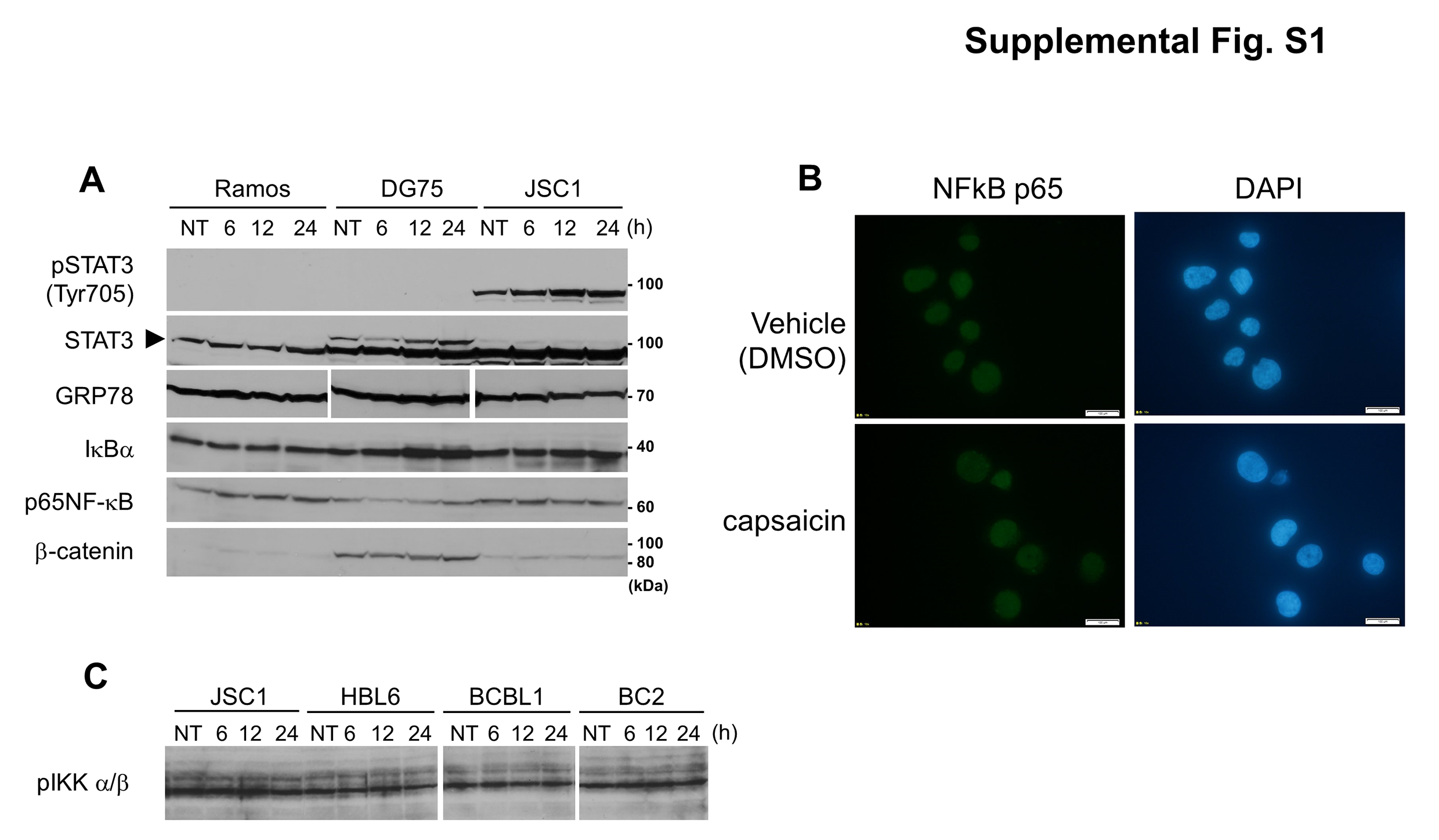

Supplement: Supplemental Figure S1 — (A) Immunoblotting analysis of the phosphorylated STAT3, GRP78, IκBα, p65NFκB, and β-catenin in capsaicin-treated B-lymphoma cells. KSHV-negative cells (Ramos and DG75) and JSC1 PEL cells were treated with 150 μM capsaicin for 6–24 h, and whole-cell lysates were assayed by immunoblotting with anti-Tyr705-phospho-STAT3, STAT3, GRP78, IκBα, p65NFκB, or β-catenin antibodies. Nontreated cells are denoted as NT. Arrowhead indicates STAT3. (B) Subcellular localization of p65NFκB in capsaicin-treated (or untreated) BCBL1 cells. BCBL1 cells were treated with capsaicin or vehicle for 6 h, the p65NFκB (green) and DNA (blue) was detected by IFA. The white bar indicates 100 μm. (C) Capsaicin did not affect phospho-IKKα/β. PEL (JSC1, HBL6, BCBL1, and BC3) cells were treated with 150 μM capsaicin for 6–24 h, and whole-cell lysates were assayed by immunoblotting with anti-phospho-IKKα/β antibodies. Nontreated cells are denoted as NT. [file Image_1.TIF]

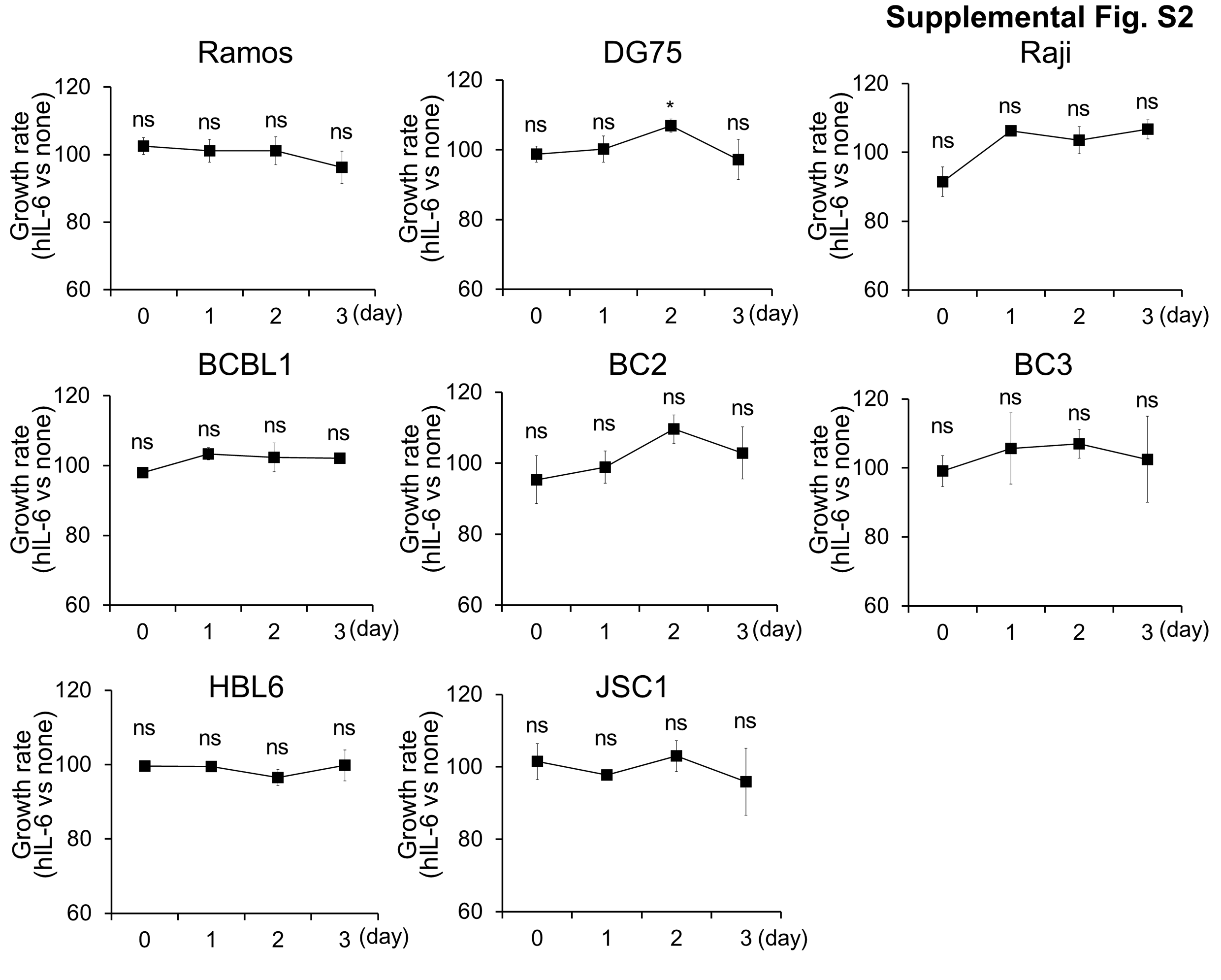

Supplement: Supplemental Figure S2 — Effect of exogenously supplied hIL-6 on proliferation of B-lymphoma cells. Cells were treated with 10 ng/mL of recombinant hIL-6 and incubated for 0–3 days. The y-axis represents the hIL-6-dependent growth rate (the cell number of the hIL-6-treated cells vs. nontreated cells). Namely, the y-axis represents the percentage of hIL-6-treated cell number when the cell number of nontreated cells on each day is defined as 100%. *P < 0.1 indicate a statistically significantly difference compared with untreated cells. ns, not significant. [file Image_2.TIF]

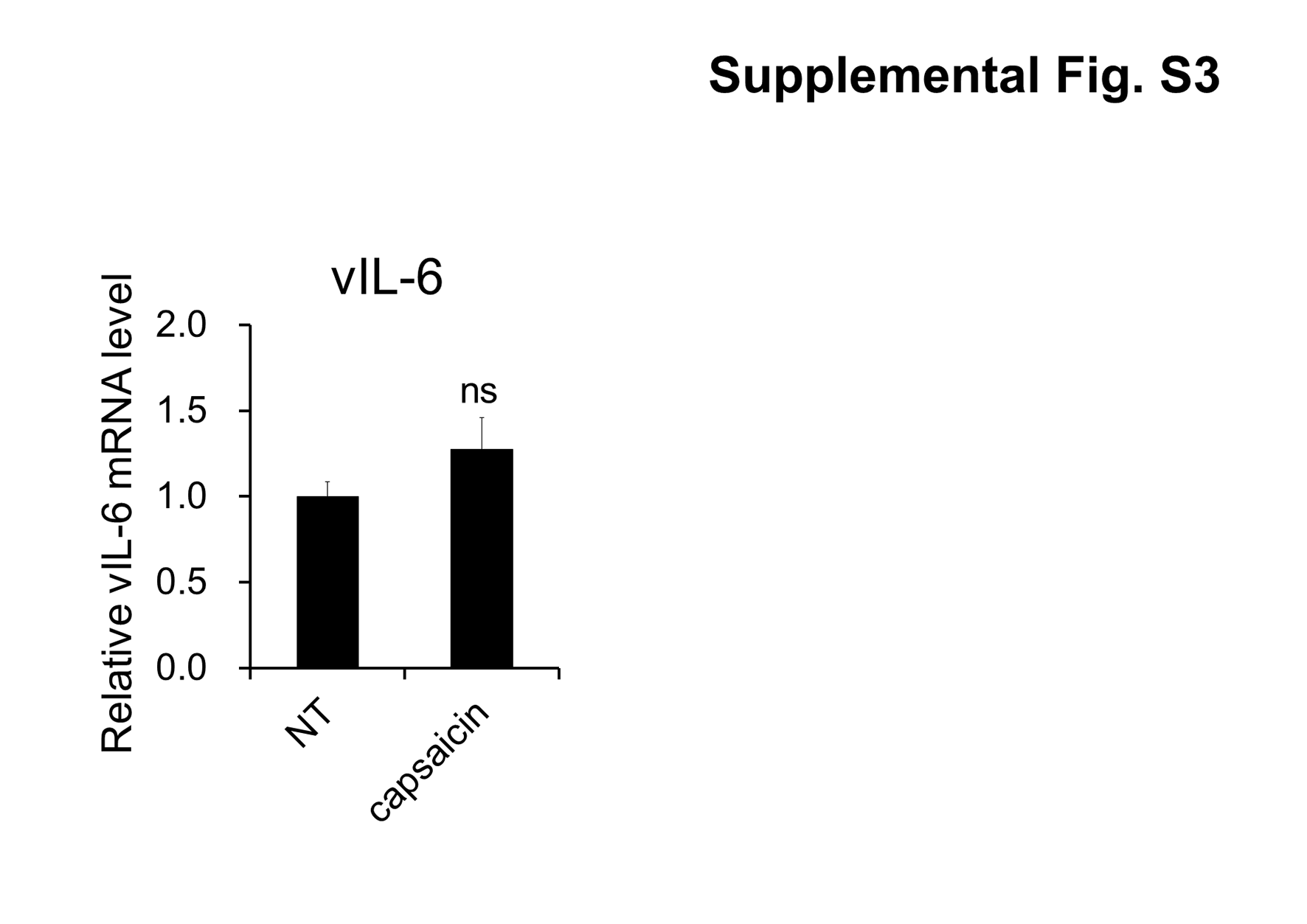

Supplement: Supplemental Figure S3 — Capsaicin treatment does not affect mRNA expression of KSHV-encoded vIL-6. BCBL1 cells were treated with 150 μM capsaicin or vehicle for 3 h, and extracted total RNA was subjected to RT-PCR to quantitate mRNA of vIL-6. The values obtained from vehicle-treated cells were defined as 1.0. ns, not significant. [file Image_3.TIF]
